# Explainable Multilayer Graph Neural Network for cancer gene prediction

**DOI:** 10.1093/bioinformatics/btad643

**Published:** 2023-10-20

**Authors:** Michail Chatzianastasis, Michalis Vazirgiannis, Zijun Zhang

**Affiliations:** LIX, École Polytechnique, IP Paris, Rte de Saclay, Palaiseau, 91120, France; LIX, École Polytechnique, IP Paris, Rte de Saclay, Palaiseau, 91120, France; Division of Artificial Intelligence in Medicine, Cedars-Sinai Medical Center, 116 N. Robertson Boulevard, Los Angeles, CA 90048, United States

## Abstract

**Motivation:**

The identification of cancer genes is a critical yet challenging problem in cancer genomics research. Existing computational methods, including deep graph neural networks, fail to exploit the multilayered gene–gene interactions or provide limited explanations for their predictions. These methods are restricted to a single biological network, which cannot capture the full complexity of tumorigenesis. Models trained on different biological networks often yield different and even opposite cancer gene predictions, hindering their trustworthy adaptation. Here, we introduce an Explainable Multilayer Graph Neural Network (EMGNN) approach to identify cancer genes by leveraging multiple gene–gene interaction networks and pan-cancer multi-omics data. Unlike conventional graph learning on a single biological network, EMGNN uses a multilayered graph neural network to learn from multiple biological networks for accurate cancer gene prediction.

**Results:**

Our method consistently outperforms all existing methods, with an average 7.15% improvement in area under the precision–recall curve over the current state-of-the-art method. Importantly, EMGNN integrated multiple graphs to prioritize newly predicted cancer genes with conflicting predictions from single biological networks. For each prediction, EMGNN provided valuable biological insights via both model-level feature importance explanations and molecular-level gene set enrichment analysis. Overall, EMGNN offers a powerful new paradigm of graph learning through modeling the multilayered topological gene relationships and provides a valuable tool for cancer genomics research.

**Availability and implementation:**

Our code is publicly available at https://github.com/zhanglab-aim/EMGNN.

## 1 Introduction

Understanding the precise function and disease pathogenicity of a gene is dependent on the target gene’s properties, as well as its interaction partners in a disease-specific context (Greene *et al.* 2015, Zitnik *et al.* 2019, Sealfon *et al.* 2021). High-throughput experiments, such as whole-genome sequencing and RNA sequencing of bulk and single-cell assays, have enabled unbiased profiling of genetic and molecular properties for all genes across the genome. Experimental methods to probe both physical (Brückner *et al.* 2009, Qin *et al.* 2021) and genetic interactions (Costanzo *et al.* 2010, Norman *et al.* 2019) provide valuable insights of the functional relevance between a pair of genes. Based on these data, computational methods have been developed to predict gene functions for understudied and uncharacterized genes by combining the gene’s property with its network connectivity patterns (Berardini *et al.* 2004, Cho *et al.* 2016a; Ietswaart *et al.* 2021, Pfeifer *et al.* 2022). However, the prediction of gene pathogenicity in disease-specific contexts is challenging. Functional assays describing the gene and its gene network are relevant to disease only to the degree to which the measured property correlates with disease physiology (Nykamp *et al.* 2017); while our understanding of complex disease physiology is poor, even for diseases with large sample sizes and data modalities, such as cancer (Liu *et al.* 2018).

As the completeness of known cancer genes is questioned, predicting novel cancer genes remains a crucial task in cancer genomics research. These genes, which are often mutated or aberrantly expressed in cancer cells, play a key role in the development and progression of the disease (Sondka *et al.* 2018). Large-scale cancer sequencing consortia projects have generated genomic and molecular profiling data for a variety of cancer types, providing an information-rich resource for identifying novel cancer genes. Building on the hypothesis that pan-cancer multi-omic modalities provide critical information to cancer gene pathogenicity, a pioneering work EMOGI (Schulte-Sasse *et al.* 2021) innovatively modeled the multi-omics features of cancer genes in protein–protein interaction (PPI) networks to predict novel cancer genes. To address the challenge of functional properties irrelevant to cancer disease physiology, EMOGI featurized each gene by a vector summarizing multi-omics data levels across various cancer types in The Cancer Genome Atlas (TCGA) (Weinstein *et al.* 2013). EMOGI then modeled the gene–gene interactions from pre-defined generic PPI networks using a Graph Convolution Neural network (GCN). When trained on a set of high-confidence cancer- and non-cancer genes, EMOGI identified 165 newly predicted cancer genes without necessarily recurrent alterations, but interact with known cancer genes.

A major limitation of EMOGI is that it did not address the disease physiology relevance in the pre-defined graph topology and connectivity patterns. EMOGI employed six different pre-defined graphs, including genetic-focused networks, such as Multinet (Khurana *et al.* 2013), and generic protein interaction networks, such as STRING-db (Szklarczyk *et al.* 2019). Among EMOGI models trained on different PPI networks, we found an average standard deviation of 25.2% in unlabeled cancer gene predictions, demonstrating the newly predicted cancer genes were different when using different PPI networks. Thus, a trustworthy adaptation of the EMOGI method’s output is challenging because conflicting prediction results are ubiquitous. Furthermore, as cancer disease physiology is complex, using a single pre-defined graph to represent the gene–gene relationships cannot fully capture its molecular landscape; therefore, more sophisticated, data-driven methods are needed to decipher the gene relationships in disease-specific contexts.

Here, we propose a novel graph-learning framework, Explainable Multilayer Graph Neural Network (EMGNN), for predicting gene pathogenicity based on multiple input biological graphs. EMGNN maximizes the concordance of functional gene relationships with the unknown disease physiology by jointly modeling the multilayered graph structure. We evaluated the performance of EMGNN in predicting cancer genes using the same compiled datasets as EMOGI and showed that our proposed method achieves state-of-the-art performance by combining information from all six PPI networks. Furthermore, we explained EMGNN’s prediction by both model-level integrated gradients (IGs) and molecular-level gene pathways. By examining newly predicted cancer genes identified by EMGNN, we demonstrated biological insights can be revealed by leveraging the complementary information in different types of biological networks. Overall, EMGNN provides a powerful new paradigm of graph learning through modeling the multilayered topological gene relationships. Our key contributions can be summarized as follows:

- We develop an EMGNN approach to identify cancer genes by leveraging multiple PPI networks and multi-omics data.
- Our method demonstrates superior performance compared to existing approaches as quantified by a significant increase in the area under the precision–recall curve (AUPRC) across six PPI networks. The average improvement in performance is 7.15% over the current state-of-the-art method, EMOGI.
- We identify the most important multi-omics features for the prediction of each cancer gene, as well as the most influential PPI networks, using model interpretation strategies.
- EMGNN identifies newly predicted cancer genes by integrating multiple PPI networks, providing a unified and robust prediction for novel cancer gene discovery. Our code is publicly available on GitHub (https://github.com/zhanglab-aim/EMGNN).

## 2 Materials and methods

### 2.1 Multilayer graph neural network

#### 2.1.1 GNNs

Let a graph be denoted by G=(V,E), where $V=\{v_{1},\ldots ,v_{N}\}$ is the set of vertices and *E* is the set of edges. Let $A\in R^{N\times N}$ denote the adjacency matrix, $X={\left[x_{1},\ldots ,x_{N}\right]}^{T}\in R^{N\times d_{I}}$ denote the node features, where *d_I_* is the features dimensions and $Y={\left[y_{1},\ldots ,y_{N}\right]}^{T}\in N^{N}$ denote the label vector. Graph neural networks typically employ a message-passing scheme, which constitutes of the two following steps. In the first step, every node aggregates the representations of its neighbors using a permutation-invariant function. In the second step, each node updates its own representation by combining the aggregated message from the neighbors with its own previous representation,


(1) $$m_{u}^{\left(l\right)}={\text{Aggregate}}^{\left(l\right)}\left(\left\{h_{v}^{\left(l-1\right)}:v\in N(u)\right\}\right),$$

(2) $$h_{u}^{\left(l\right)}={\text{Combine}}^{\left(l\right)}\left(h_{u}^{\left(l-1\right)},m_{u}^{\left(l\right)}\right),$$

where $h_{u}^{\left(l\right)}$ represents the hidden representation of node *u* at the lth layer of the GNN architecture. Many choices for the Aggregate and Combine functions have been proposed in recent years, as they have a huge impact on the representation power of the model (Xu *et al.* 2019). Among the most popular architectures, are GCNs (Kipf and Welling 2017), and Graph Attention Networks (GAT) (Velikovi *et al.* 2018). In GCN, each node aggregates the feature vectors of its neighbors with fixed weights inversely proportional to the central and neighbors node degrees, $h_{u}^{\prime }=W^{⊤}\sum_{v\in N(u)\cup \{u\}}\frac{h_{v}}{\sqrt{{\hat{d}}_{v}{\hat{d}}_{u}}}$, with ${\hat{d}}_{i}=1+\sum_{j\in N(i)}1$. In GAT, each node aggregates the messages from its neighbor using learnable weighted scores: $h_{u}^{\prime }=\alpha_{u,u}Wh_{u}+\sum_{v\in N(u)}\alpha_{u,v}Wh_{v}$, where the attention coefficients $\alpha_{u,v}$ are computed as


$$\alpha_{u,v}=\frac{\;\text{exp}\;\left(\text{LeakyReLU}\left(a^{⊤}[Wh_{u}\;||\;Wh_{v}]\right)\right)}{\sum_{k\in N(u)\cup \{u\}}\;\text{exp}\;\left(\text{LeakyReLU}\left(a^{⊤}[Wh_{u}\;||\;Wh_{k}]\right)\right)}.$$

#### 2.1.2 Multilayer graph construction

Extending graph neural networks to handle multiple networks is not a trivial task, as they are designed to operate on a single graph. Next, we describe our method, which can accurately learn node representations using graph neural networks, from multilayer graphs.

Let *N* be the total number of genes, each associated with a feature vector $x_{j}\in R^{d_{I}}$. Let also *K* be the number of gene–gene interaction networks. We represent each network $G^{\left(i\right)}$ with an adjacency matrix $A^{\left(i\right)}\in Z^{N_{i}\times N_{i}}$ and feature matrix $X^{\left(i\right)}\in R^{N_{i}\times d_{I}}$, where *N_i_* is the number of genes in the *i*-th network. Since some genes are not presented in all the graphs, the following equation holds $N_{i}\leq N,\;i\in \{0,1,\ldots ,K-1\}$.

In the first step, for each graph *G^(i)^*, we apply a graph neural network *f*_1_ that performs message-passing and updates the node representation matrix $H^{\left(i\right)}=f_{1}(X^{\left(i\right)},A^{\left(i\right)})$ of each graph i∈{0,1,…,K−1}. We set *f*_1_ to be shared across all graphs. This design allows us to handle a variable number of graphs while keeping the number of trainable parameters fixed.

**Figure 1.**
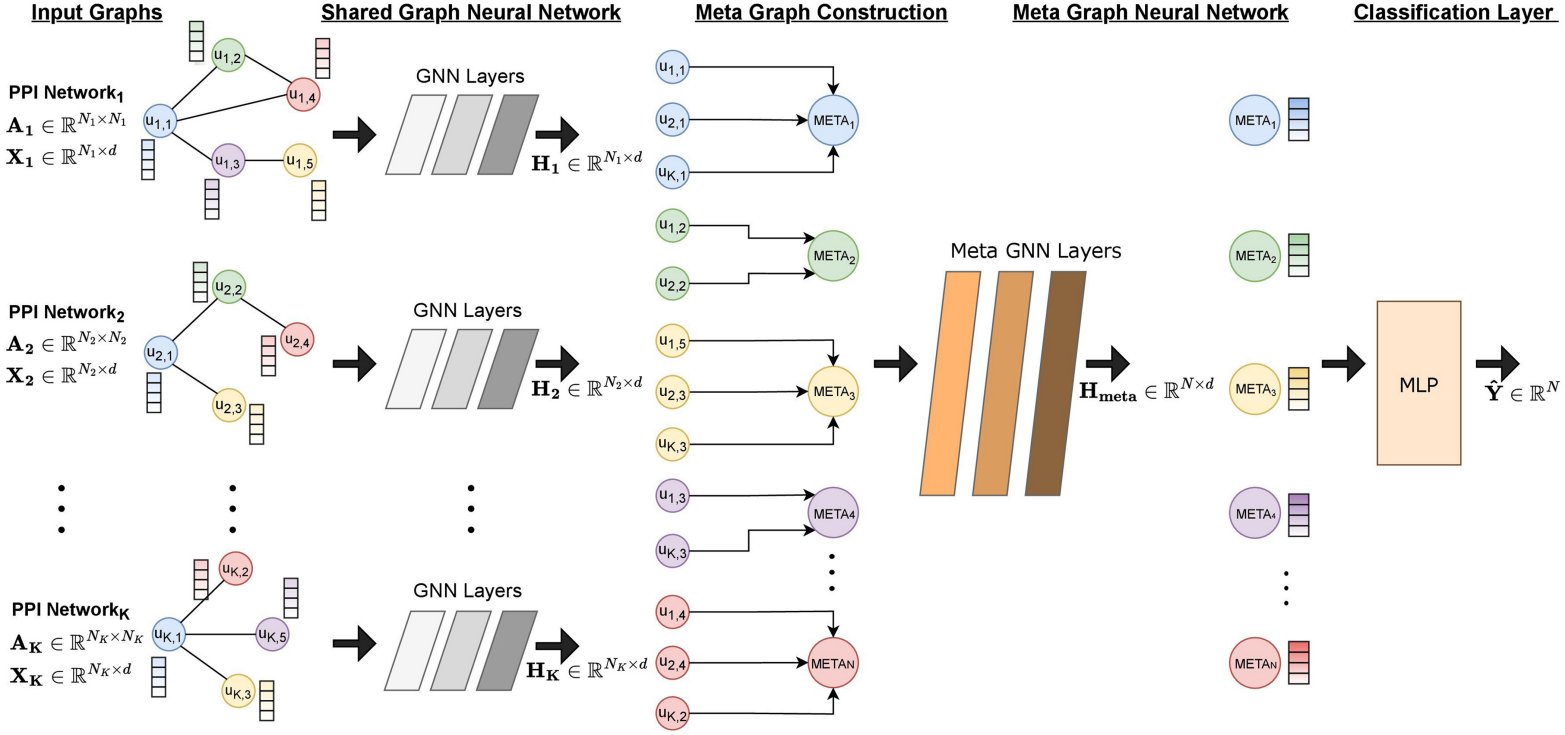
An illustration of our proposed EMGNN approach. The model consists of three main steps: (i) apply a shared GNN to update the node representation matrix of each input graph; (ii) construct a meta graph for each gene, where the same genes across all graphs are connected to a meta node, and update the representation of the meta nodes with a second GNN, Meta GNN; and (iii) use a multilayer perceptron to predict the class of each meta node.

Next, to aggregate and share information between each graph, we construct a meta graph $G_{\mathrm{meta},j}$ for each gene/node *j*, where the same genes *j* across all graphs are connected to a meta node *v_j_*. We initialize the features of the meta node *v_j_* with the initial features of the corresponding gene *j*. We apply a second GNN *f*_2_ to update the representation of the meta node *v_j_*, $H_{\mathrm{meta},j}=f_{2}(X_{\mathrm{meta},j},A_{\mathrm{meta},j})$, where $X_{\mathrm{meta},j}$ contains the features of gene *j* from all the networks and $A_{\mathrm{meta},j}$ is the adjacency matrix of the meta graph $G_{\mathrm{meta},j},\;j\in \{0,1,\ldots ,N\}$. We set *f*_2_ to be shared across all genes. Therefore, in this stage, the model combines and exchanges information between the different networks. Finally, a multilayer perceptron *f*_3_ predicts the class of the meta node *j*, $\hat{y_{j}}=f_{3}(H_{\mathrm{meta},j})$. An illustration of the proposed model can be found in Fig. 1.

#### 2.1.3 Datasets

We use the datasets and train/test splits compiled by Schulte-Sasse *et al.* (2021) to ensure a fair comparison. Specifically, we trained our proposed model with six PPI Networks: CPDB (Kamburov *et al.* 2011), Multinet (Khurana *et al.* 2013), PCNet (Huang *et al.* 2018), STRING-db (Szklarczyk *et al.* 2019), Iref (Razick *et al.* 2008), and its newest version Iref(2015). We provide a brief description of the preprocessing steps here for clarity and self-containment. Depending on the source of the data, different confidence thresholds were applied to filter out low-confidence interactions. Interactions with a score higher than 0.5 in CPDB and 0.85 in STRING-db were included. For Multinet and IRefIndex (2015), the data were obtained from the Hotnet2 GitHub repository (Reyna *et al.* 2018). In the case of the recent version of IRefIndex, analysis was restricted to binary interactions between two human proteins. No further processing was performed on the PCNet.

As node features, we used gene mutation frequencies (MF), copy number aberrations (CNA), DNA methylation (METH), and gene expression (GE) data of 29 446 samples from TCGA (Weinstein *et al.* 2013), from 16 different cancer types. The single-nucleotide variation (SNV) frequency was calculated for each gene in each cancer type by dividing the number of non-silent SNVs in that gene by its exonic gene length. Gene-associated CNAs were collected from TCGA, where the copy number rate for each gene was defined as the number of times it was amplified or deleted in a specific cohort. DNA methylation for each gene in a cancer type was computed as the difference in methylation signal between tumor and matched normal samples. The expression level of each gene in each sample was quantified using RNA-seq data (Wang *et al.* 2018), and differential expression was computed as a ${\;\text{log}\;}_{2}$ fold change between cancer and matched normal samples, averaged across samples.

The positive examples included expert-curated cancer genes, high-confidence cancer genes mined from PubMed abstracts, and genes with altered expression and promoter methylation in at least one cancer type (Sondka *et al.* 2018, Repana *et al.* 2019). The negative examples were compiled by removing genes not associated with cancer from a set of all genes and were selected based on their absence in the positive set and various cancer databases (McKusick 2007, Repana *et al.* 2019). For further information regarding the data collection and processing methods, refer to the study by Schulte-Sasse *et al.* (2021).

#### 2.1.4 Experimental details

To ensure a fair comparison with previous work, we utilized the same experimental setup as Schulte-Sasse *et al.* (2021). In particular, we divided the data for each testing graph into a 75% training set and a 25% testing set using stratified sampling to maintain equal proportions of known cancer and non-cancer genes in both sets. Since our model takes multiple graphs as input for each experiment, we retained the test nodes of one graph as the test set, and we allocated 90% of the remaining nodes from the other graphs to the training set and 10% to the validation set. When adding other PPI networks to the training and validation set, we held out the same testing set from the combined training set and kept the test set identical to previous works (Schulte-Sasse *et al.* 2021, Hong *et al.* 2022). An illustration of the process of adding a new biological network and a definition of training and validation splits are shown in Supplementary Fig. S1. The hyperparameters we tuned include the learning rate, weight decay, the number of hidden units, the number of attention heads for the GAT layers, the dropout rate, and the number of layers. The model was trained for 2000 epochs, using the cross-entropy loss function, and the ADAM optimizer (Kingma and Ba 2015) with a learning rate of 0.001. The initial GNN had three layers with a hidden dimension of 64, while the meta-GNN had a single layer with a hidden dimension of 64. The Supplementary Table S5 provides information on the PPI networks, including statistics, as well as the number of positive and negative genes used for training. We provide a description of all the baselines in the Supplementary Material.

### 2.2 Model interpretation

We used the IG module in Captum, to assign an importance score to each input feature. Captum is a tool for understanding and interpreting the decision-making process of machine-learning models (Kokhlikyan *et al.* 2020). The IG approximates the integral of gradients of the model’s output with respect to the inputs along a straight line path from a specific baseline input to the current input. The baseline input is typically chosen to be a neutral or a meaningless input, such as an all-zero vector or a random noise. Formally, let *F*(*x*) be the function of a neural network, where *x* is the input and $\hat{x}$ is the baseline input. The IGs for input *x* and baseline *x*_0_ along the *i*-th dimension is defined as: $\mathrm{IntegratedGrad}s_{i}(x)=(x_{i}-\hat{x_{i}})\int_{\alpha =0}^{1}\frac{\partial F(\hat{x}+\alpha (x-\hat{x})}{\partial x_{i}}d\alpha$, where the integral is taken along the straight line path from $\hat{x}$ to *x* and $\partial F(x)/\partial x_{i}$ is the gradient of *F*(*x*) along the *i*-th dimension.

However, the traditional IG method, which is designed for single input models, is not directly applicable to graph neural networks as they have two distinct inputs, namely node features and network connectivity. This necessitates the development of a modified approach for computing IGs in graph neural networks that considers both inputs. To this end, we propose a decomposition of the problem into two parts: identifying the most important node features and identifying the most crucial edges in the network separately. Since we predict the class of each gene by combining all the graphs, from the meta node representations, we apply the interpretation analysis only to the meta nodes.

#### 2.2.1 Node feature interpretation analysis

We analyse the contribution of node features to the predictions of the GNN by using the traditional IG method while keeping the edges in the network fixed. Specifically, we interpolate between the current node features input and a baseline input where the node features are zero: $\mathrm{Attributio}n_{x_{i}}=(x_{i}-\hat{x_{i}})\int_{\alpha =0}^{1}\frac{\partial F(\hat{x}+\alpha (x-\hat{x},A)}{\partial x_{i}}d\alpha$, where *A* are the adjacency matrices of the graphs. Since the prediction for each gene is also based on the features of surrounding genes in the graphs, we extract attribution values for the *k*-hop neighbor genes as well, where *k* is equal to the number of message-passing layers in the first GNN. Therefore, the output of the attribution method for each node *u*, is a matrix $K^{\left(u\right)}\in R^{N\times d}$. Each entry *K_ij_* of the matrix, corresponds to the attribution of the feature *j* of node *i* to the target node *u*. From this matrix, we select the row that corresponds to the feature attributions of the corresponding meta node.

#### 2.2.2 Edge feature interpretation analysis

To analyse the contribution of edges in the meta graph to the predictions of the GNN, we use the IG method for the edges while keeping the node features fixed. Specifically, we interpolate between the current edge input and a baseline input where the weights of the edges are zero: $\mathrm{Attributio}n_{e_{i}}=\int_{\alpha =0}^{1}\frac{\partial F(X,A_{\alpha })}{\partial w_{e_{i}}}d\alpha$, where $A_{\alpha }$ corresponds to the graphs with the edge weights equal to *α*. We further normalize the attribution values of each meta node by dividing them by their maximum value, resulting in a range of [0, 1] for each edge. This explanation technique allows us to understand which edges in the meta graph are crucial for the model’s decision-making process, and therefore which input PPI networks are important for each gene prediction.

### 2.3 Newly predicted cancer gene discovery

We applied the trained EMGNN model that combined all six individual PPI networks to predict novel cancer genes in the 14 019 unlabeled genes. We ranked these genes by their predicted cancer gene probability for potential new predicted cancer genes in this study. For each unlabeled gene, we also applied EMOGI models trained on individual PPI networks to predict the probability of it being a cancer gene. The complete list of the predictions for all the unlabeled genes, can be found in the project repository on GitHub.

### 2.4 Gene set enrichment analysis

To understand the biological mechanisms of EMGNN’s cancer gene prediction, we employed gene set enrichment analysis (GSEA) to analyse the functional enrichment of important gene features in curated cancer pathway annotations. Specifically, to determine the importance of neighboring gene nodes, we aggregated the maximum feature importance of each node using Captum’s feature explanation results. Genes with zero importance were excluded in this analysis as they did not contribute to the prediction of this target gene. We then ranked the neighboring gene nodes based on their importance and used this ranked gene list as input for GSEA. The enrichment *P*-value and multiple testing corrected FDR were computed by GSEA python package (Fang *et al.* 2023) against cancer hallmark gene sets (Liberzon *et al.* 2015).

## 3 Results

### 3.1 Multilayered graph improves EMGNN performance

To effectively model multilayered graph structures for cancer gene prediction, we developed EMGNN, a graph neural network model that jointly learned from multiple biological networks as inputs (Fig. 1). We applied EMGNN to predict cancer genes due to the wealth of multi-omic profiling data available for cancer, yet the underlying tumorigenesis is highly complex and cannot be fully captured by a single biological network. To ensure fair comparisons, we used a compiled dataset and kept the identical training/test split from a previous report (Schulte-Sasse *et al.* 2021) (Section 2). In total, this dataset consisted of 887 labeled cancer genes, 7753 non-cancer genes, and 14 019 unlabeled genes, along with 6 PPI networks with multi-omics features. The detailed numbers of labeled positive and negative genes for training the EMGNN model in each PPI network can be found in Supplementary Table S5.

**Figure 2.**
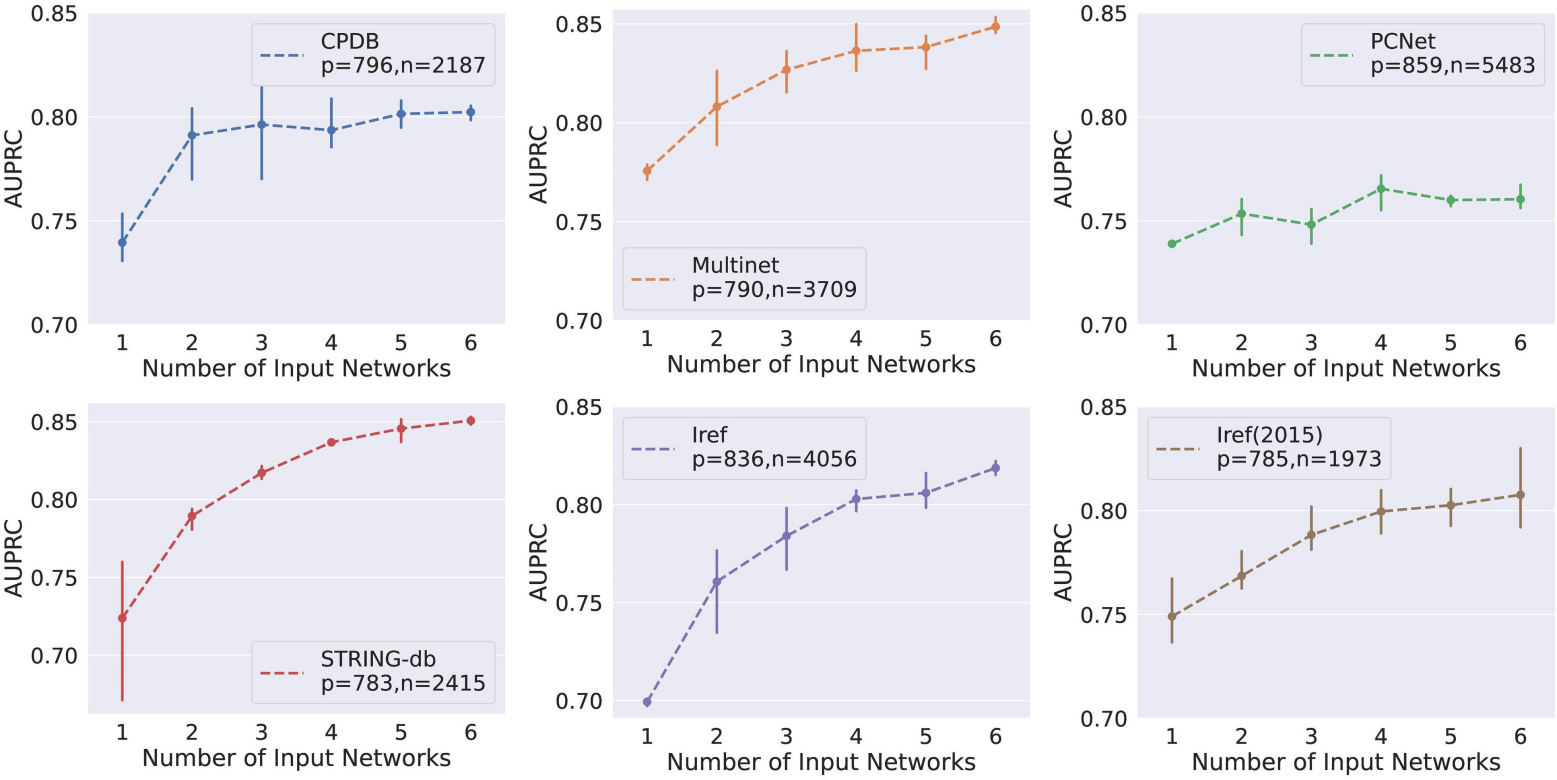
Test AUPRC and standard deviation values of EMGNN(GCN) with respect to the number of input PPI networks. Each line represents a test set of positive and negative labeled genes held out in a specific PPI network. The addition of PPI networks was conducted using a random sampling approach, where three combinations of PPI networks were sampled randomly at each point. Note that the testing nodes remain the same as more networks are added. We observe that the performance increased for the majority of the test datasets, as the number of input networks increases.

The integration of multiple graphs leads to substantial improvements in the performance of EMGNN. We trained EMGNN models and evaluated the testing performance with respect to different numbers of PPI networks. While we added other networks to the training and validation set, we held out the same testing set from the combined training set and kept the test set identical to previous works (Schulte-Sasse *et al.* 2021, Hong *et al.* 2022). An illustration of the process of adding a new biological network and a definition of training and validation splits are shown in Supplementary Fig. S1. As shown in Fig. 2, the testing performance increased for each of the six testing datasets as the number of input networks increased.

EMGNN trained by incorporating all six graphs achieved state-of-the-art performance for all six test sets (Table 1). As each test set was an independent set of held-out labeled cancer and non-cancer genes from each network and we kept the set identical to previous reports (Section 2), the testing performance will inform generalization error of the trained EMGNNs and provide fair comparisons to previous results. EMGNN outperformed the current state-of-the-art method EMOGI by an average margin of 7.15% AUPRC across all test sets, with the largest gain of 11.1% in performance observed in the old version Iref. For PCNet test set where the gain was the smallest, EMGNN combining six graphs is still significantly more accurate than EMOGI using PCNet (*P*-value = .012, *t*-test). Importantly, we show that the performance gain by combining all six PPI networks in EMGNN could not be achieved by a simple ensemble method, such as a majority vote (Table 1). Therefore, incorporating information from multiple networks leads to enhanced predictive power for gene pathogenicity prediction. In addition, we extended our analysis beyond traditional PPI networks to include tissue-specific networks. By leveraging biological networks originating from three diverse tissue types, namely the adrenal cortex, adipose tissue, and blood, we sought to assess EMGNN’s adaptability to tissue-specific intricacies in cancer genomics. Detailed results of this experiment can be found in the Supplementary Table S4, demonstrating EMGNN’s proficiency in predicting cancer genes using tissue-specific functional networks. We consistently observe that the integration of multiple networks continues to enhance the model’s predictive capabilities for gene pathogenicity prediction for both PPI and tissue-specific functional networks.

**Table 1.** Test AUPRC values and standard deviation across different PPI networks across five different runs.

Method	CPDB	Multinet	PCNet	STRING-db	Iref	Iref(2015)
Random	0.27	0.18	0.14	0.24	0.17	0.28
20/20+	0.66	0.62	0.55	0.67	0.61	0.65
MutSigCV	0.38	0.33	0.27	0.41	0.35	0.43
HotNet2 diffusion	0.62	0.56	0.48	0.50	0.45	0.65
DeepWalk+features RF	0.74	0.71	0.72	0.71	0.66	0.71
PageRank	0.59	0.53	0.54	0.44	0.42	0.62
GCN without omics	0.57	0.53	0.47	0.39	0.37	0.64
DeepWalk + SVM	0.73	0.51	0.63	0.52	0.62	0.66
RF	0.60	0.59	0.51	0.61	0.54	0.62
MLP	0.58	0.63	0.47	0.63	0.55	0.64
EMOGI(Schulte-Sasse *et al.* 2021)	0.74	0.74	0.68	0.76	0.67	0.75
EMOGI(Majority Vote)	0.768	0.783	0.673	0.781	0.676	0.775
EMOGI(Hong *et al.* 2022)	0.775 ± 0.003	0.732 ± 0.003	0.745 ± 0.002	0.763 ± 0.003	0.701 ± 0.004	0.757 ± 0.001
EMGNN(GCN)	**0.809 ±**0.006	**0.854 ±**0.007	**0.761 ±**0.001	**0.856 ±**0.002	**0.822 ±**0.002	**0.800 ±**0.010
EMGNN(GAT)	0.776 ± 0.018	0.796 ± 0.034	0.730 ± 0.031	0.805 ± 0.030	0.739 ± 0.033	0.773 ± 0.049

Bold numbers indicate the best results.

### 3.2 Evaluating the performance of different GNN architectures and ablations

To understand each model component's contribution to EMGNN’s superior performance and model robustness, we next performed an ablation study using different GNN architectures and input perturbations. For GNN architectures, we compared GCN (Kipf and Welling 2017), and GAT (Velikovi *et al.* 2018). After identifying the most effective GNN architecture, we proceeded to evaluate its performance under varied input conditions, including the incorporation of random and constant features, and the random removal of 20% and 40% of edges within the input graphs.

As shown in Table 1, the GNN architecture played an essential role in EMGNN testing performance. We observed that GCN is the best-performing GNN architecture in all test datasets. Our findings demonstrated that the choice of GNN architecture has a significant impact on the performance of our model. Thus, EMGNN refers to EMGNN(GCN) throughout the article unless otherwise stated.

Biologically, the EMGNN node features and edges are determined using high-throughput assays that inherently have measurement errors. To this end, we sought to answer the two following questions: how robust is EMGNN to node feature and graph structure perturbations? Are the node features and the graph structure both crucial for the prediction of cancer genes? We examined the performance of EMGNN under different types of input perturbations (Supplementary Table S1), including the removal of the multi-omics node features and edges, and the addition of random or constant vectors.

We found that EMGNN decreased in performance for both random and all-one node features, suggesting that the node features derived from TCGA consortia were informative and highly relevant to cancer pathophysiology and cancer gene pathogenicity. For edge ablations, we randomly removed 20% and 40% edges in each PPI network. The removal of edges slightly decreased EMGNN performance. This is expected because EMGNN integrates six PPI networks and demonstrates its robustness toward connectivity patterns by jointly modeling a multilayered graph topology. Overall, EMGNN effectively leveraged both node features and edges to achieve accurate predictions. We further performed additional ablation studies to examine the impact of various hyperparameters, such as the number of GNN layers (Supplementary Table S2) and the confidence threshold of the edges for constructing the PPI networks (Supplementary Table S3). We observed notable improvement in the performance by increasing the number of layers from 1 to 3, suggesting that a deeper architecture can capture more intricate gene–gene interactions within the network. We report the results in the Supplementary Material.

### 3.3 Explaining EMGNN reveals biological insights of cancer gene pathogenicity

Explainable and trustworthy models are essential for understanding the biological mechanisms of known cancer genes and facilitating the discovery of novel cancer genes. Given EMGNN’s accurate and robust predictive performance, we developed Integrated Gradients methods (Kokhlikyan *et al.* 2020) to explain the node and edge attributions of EMGNN (Section 2).

**Figure 3.**
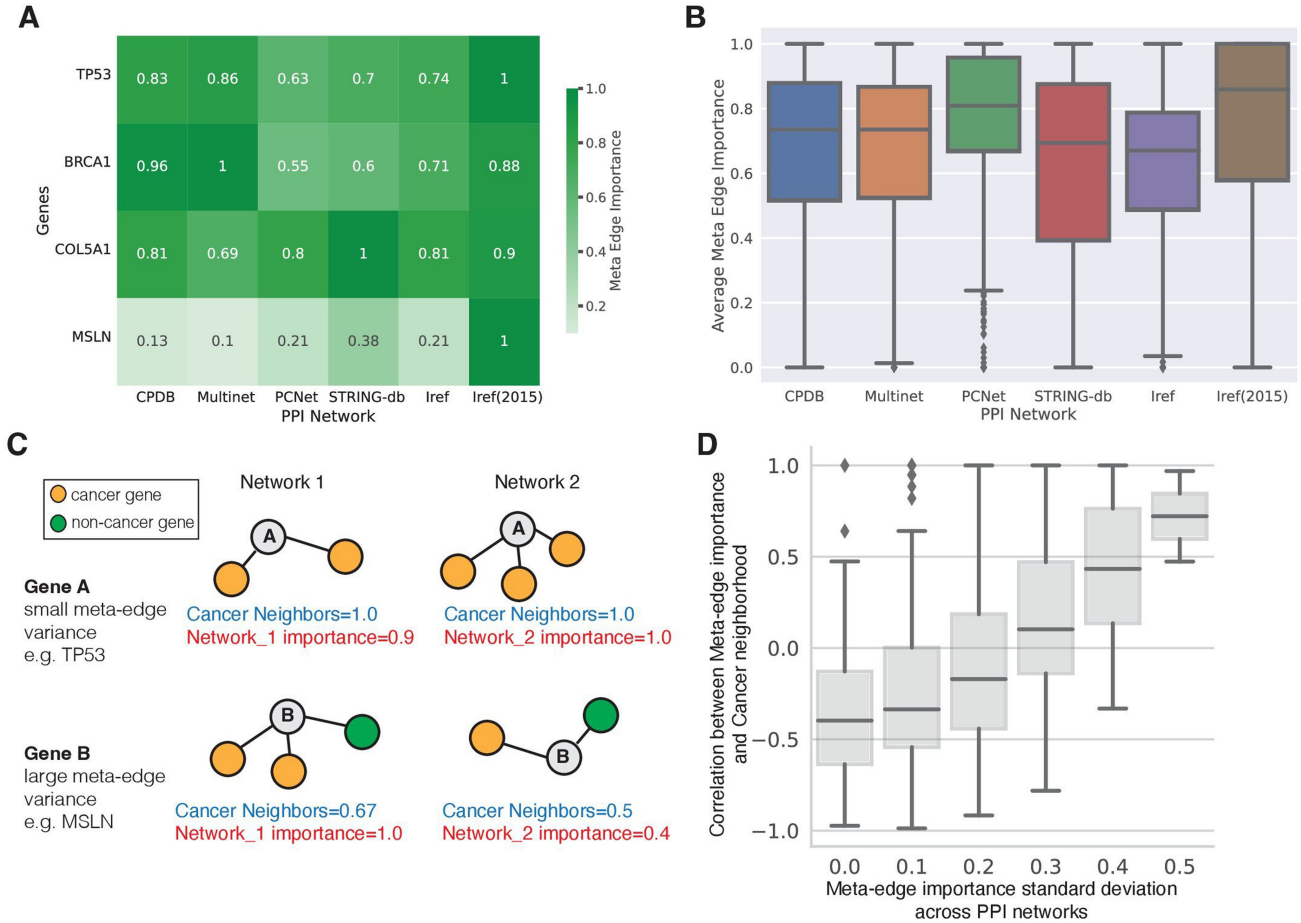
Explanation of each PPI network’s contribution to cancer gene predictions. (A) Representative PPI network contributions in known cancer genes and newly predicted cancer genes. TP53 and BRCA1 are known cancer genes; COL5A1 and MSLN are newly predicted cancer genes. (B) Overall distribution of meta-edge feature importance for all known cancer genes across six PPI networks. Meta-edge feature importance was normalized to one (see Section 2 for details). (C) A hypothetical illustration of PPI network cancer neighborhood implicated in the variation of meta-edge importance. (D) Empirical analysis demonstrates a higher correlation between meta-edge importance and cancer neighborhood for genes with a large meta-edge variance.

A unique advantage of EMGNN is its integration of multiple PPI networks; therefore, we focused our analysis on the relative contributions from each PPI network to the known cancer gene predictions (Fig. 3A). Each PPI network’s importance was measured by the corresponding meta-edge importance (Section 2). We examined the relative contributions for two well-known cancer genes (TP53 and BRCA1, predicted cancer gene probability $\hat{y}=0.99,0.98$, respectively) and two newly predicted cancer genes (COL5A1 and MSLN, predicted cancer gene probability $\hat{y}=0.98,0.90$, respectively; see Fig. 3A). Notably, different genes were predicted as cancer genes leveraging evidence from different PPI networks. For example, Multinet contributed to BRCA1, but not for MSLN. The newly predicted cancer gene COL5A1 combined information from all six PPI networks. To systematically examine if some PPI networks were statistically more important contributors than others, we performed an ANOVA test across all meta-edge importance for cancer genes (Fig. 3B). We found that different PPI networks made significantly different contributions to cancer gene prediction (*P*-value = 1.2e−65; ANOVA). This suggests that certain PPI networks were more informative than others, likely due to their unique connectivity patterns that were more reflective of cancer development and progression. Thus, EMGNN successfully learned complementary information from the connectivity patterns in each graph, as shown by the overall contributions from individual graphs and gene-specific variations.

We hypothesized that the variation of meta-edge importance was a result of different cancer gene neighborhood in different PPI networks (Fig. 3C). For a target gene, we define “cancer neighbors” as the number of neighboring genes that are known cancer genes, which is then normalized by the degree of the target node in the given network. Hypothetically, for gene A whose neighbors were all cancer genes across PPI networks, the meta-edge importances were also comparable. In contrast, for gene B whose cancer gene neighbors varied across PPI networks, we should observe a positive covariance between cancer gene neighbors and meta-edge importance. To test our hypothesis, we computed the standard deviations of meta-edge importance for each labeled cancer gene, and the Pearson correlation between meta-edge importance and the cancer gene neighbors across PPI networks. For genes with large standard deviation of meta-edge importance, the important PPI networks tend to have more cancer neighbors, as demonstrated by a higher correlation between meta-edge importance and cancer neighbors (Fig. 3D). Due to the complex graph convolutions that enabled message-passing beyond one-hop neighbors, our cancer neighbors may not fully capture the variations of PPI network importance. Overall, our empirical analysis demonstrates that cancer neighborhood is implicated in genes with divergent connectivity patterns across PPI networks.

**Figure 4.**
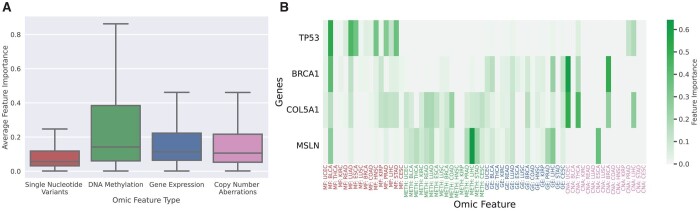
Explanations of multi-omic node feature importance in cancer gene predictions. (A) Overall distribution of node feature importance grouped by omic feature types, including single-nucleotide variants (MF), DNA methylation (METH), gene expression (GE), and copy number aberrations (CNA), for known cancer genes. (B) Detailed node feature importance for the four genes analysed in Fig. 3B. *X*-axis labels were color-coded to match the omic feature types in (A). Individual tumor types were coded according to TCGA study abbreviations (Weinstein *et al.* 2013).

On the individual gene level, a detailed explanation will reveal gene-specific genetic and molecular aberrations that the EMGNN model relies on for cancer gene prediction. As EMGNN’s node features were derived by multi-omics pan-cancer datasets, we next assessed if certain types of omic data were informative to cancer gene predictions. Our model explanation results of node features indicated that SNV features were found to be significantly less informative than other types of features, which is consistent with previous reports that CNAs were more detrimental to cancer progression than SNVs (Elmarakeby *et al.* 2021). In contrast, we observed that DNA methylation was significantly more important for known cancer gene prediction than other omics data (*P*-value <.01 for all three pairwise *t*-test of other omics against DNA methylation). We further examined the node feature importance of the same four genes (TP53, BRCA1, COL5A1, and MSLN). As expected, the omics feature contributions varied on different genes and were highly gene specific, demonstrating the heterogeneity of tumorigenesis. Point mutations were major contributors to the prediction of TP53 as a cancer gene, which is consistent with findings from previous studies (Guimaraes and Hainaut 2002, Almeida *et al.* 2009). Moreover, the prediction of BRCA1 correctly identified gene expression and copy number aberrations as the most significant features (Chang *et al.* 2022). DNA methylation had a moderate contribution for the two newly predicted cancer genes, COL5A1 and MSLN (Fig. 4B). As DNA methylations are reversible epigenetic modifications, this may suggest potential novel therapeutic targets for certain cancer genes mediated by DNA methylation (Sharma *et al.* 2010).

### 3.4 EMGNN identifies newly predicted cancer genes by integrating multilayer graphs

A key utility of EMGNN, given its superior performance and explainability, is to identify newly predicted cancer genes that share similar topological patterns to known cancer genes, but may have been missed by a conventional recurrent alteration analysis (Schulte-Sasse *et al.* 2021, Sherman *et al.* 2022). By integrating multilayer graphs, EMGNN addressed the divergent and inconsistent cancer gene predictions from previous models trained using a single PPI network. Prior to EMGNN, models trained using single PPI networks, such as EMOGI, had made conflicting predictions on which genes were cancerous. Indeed, we observed substantial variations among the predictions of EMOGI models trained on individual PPI networks, with an average of 29% and 63% difference between the highest and lowest predictions for the top-100 and all unlabeled nodes, respectively. Furthermore, we found an average standard deviation of 25.2% in unlabeled cancer gene predictions of EMOGI, demonstrating the predicted novel cancer genes were different when using different PPI networks. In contrast, EMGNN resolved these discrepancies using a more accurate and robust representation of the data from multilayer graphs.

Our analysis identified 435 genes with a high probability of being newly predicted cancer genes with a precision threshold at 95%. The identification of these novel cancer genes may provide new insights into the molecular mechanisms of cancer and offer potential targets for the development of novel therapies, demonstrating that machine-learning predictions can augment the completeness of cancer gene catalogs (Schulte-Sasse *et al.* 2021).

**Figure 5.**
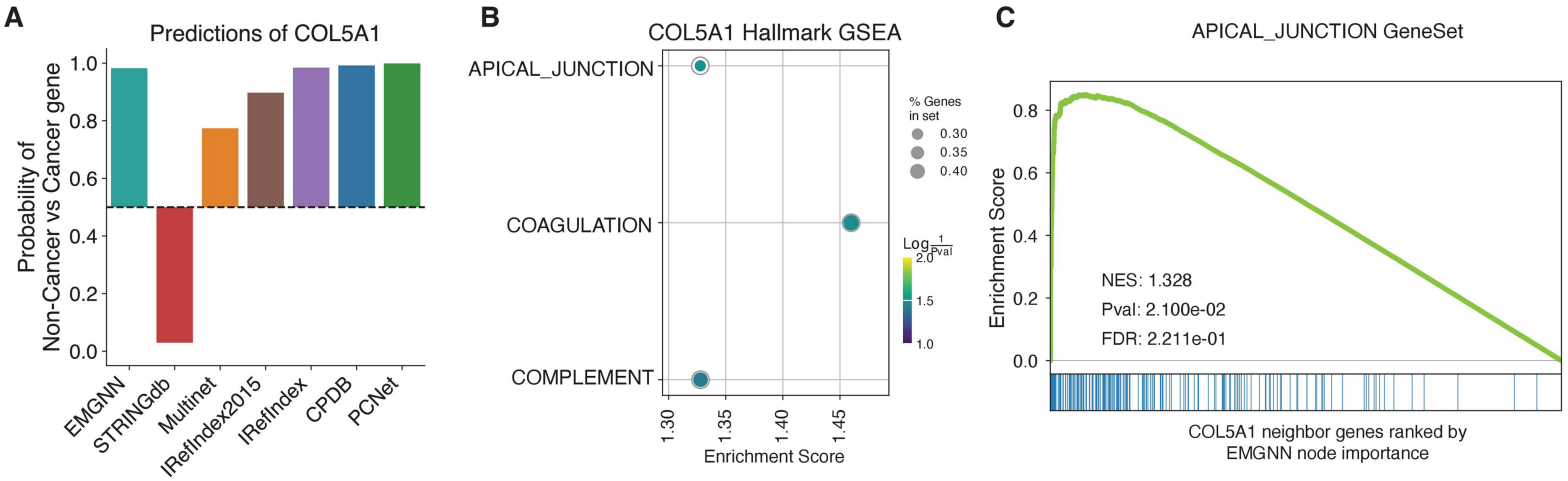
EMGNN predicts COL5A1 as a novel cancer gene and reveals biological insights. (A) A comparison of predicted cancer gene probability from EMGNN and EMOGI models trained on single PPI networks. As a probability of 50% equaled random guessing between cancer versus non-cancer gene, the bar heights reflected the prediction confidence. (B) Three cancer hallmark genesets were significantly enriched in the important neighboring genes of COL5A1 as revealed by interpreting EMGNN model. (C) Enrichment of apical junction cancer hallmark geneset in COL5A1 neighboring genes. The neighboring genes of COL5A1 were ranked by their EMGNN node importance on the *x*-axis, with each bar representing a gene in the apical junction geneset. A strong left-shifted curve demonstrates enrichment of apical junction geneset in the top important genes to predict COL5A1 as a cancer gene.

As a case study, we analysed the predictions of a newly predicted cancer gene, COL5A1 (Fig. 5). For this gene, EMOGI model trained on STRINGdb predicted a non-cancer gene with high confidence ($\hat{y}=0.03$), EMOGI models trained on IRefIndex, CPDB, and PCNet predicted a cancer gene with high confidence ($\hat{y}>0.98$), while the models trained on Multinet and IRefIndex2015 predicted a cancer gene with moderate likelihood ($\hat{y}=0.775$ and 0.897, respectively). This level of divergence in predictions hinders a trustworthy adaptation of model predictions in clinical and pragmatic settings. In contrast, EMGNN integrated the information from each individual PPI network in a data-driven approach and provided more accurate, unified predictions of cancer genes (Table 1). EMGNN predicted COL5A1 as a cancer gene with high confidence (Fig. 5A). Importantly, we also found all individual PPI networks were contributing similarly to the final EMGNN prediction (Fig. 3A), and revealed the multi-omics features implicated in this prediction (Fig. 4B).

Leveraging the explanation results of node contributions for COL5A1 across its neighboring genes, we further illustrated the potential biological mechanisms of COL5A1 using a GSEA (Section 2). These neighboring genes formed a ranked gene list based on their explained EMGNN contributions to the prediction of COL5A1 as a cancer gene or not. We discovered that three cancer hallmark gene sets, i.e. apical junction, coagulation, and complement system (part of the innate immune system), were significantly enriched in COL5A1 neighboring genes (Fig. 5B). For example, the apical junction cancer hallmark geneset contained genes annotated to function in cell–cell adhesion among epithelial cells, many of those were enriched in the top contributors (Fig. 5C). This was further supported by other studies, where COL5A1 was associated with skin cancer, the type of cancer with a strong epithelial cell origin (Mann *et al.* 2015, Gu *et al.* 2021, Zhu *et al.* 2022). Therefore, we demonstrated how molecular mechanisms of newly predicted cancer genes could be interpreted and discovered using the explainable EMGNN framework.

## 4 Discussion

The biomedical and biological domain contains a wealth of information, which is often represented and analysed using graph structures to reveal relationships and patterns in complex data sets. Various gene interaction and PPI networks describe the functional relationships of genes and proteins, respectively. The gene–gene relationships were often described in generic cellular contexts and/or by integrating different, heterogeneous sources of information. Therefore, a single graph often struggles to best match disease-specific conditions, and different graph construction and integration methods render distinct predictive powers (Cao *et al.* 2014, Hristov and Singh 2017, Horn *et al.* 2018). Substantial efforts have been devoted to develop integrated (Cao *et al.* 2014, Cho *et al.* 2016b) and tissue-specific graphs (Zitnik and Leskovec 2017). Here, we took a complementary approach and developed a new graph-learning framework, EMGNN, to jointly model numerous graphs. Applying EMGNN to predict cancer genes demonstrated its superior performance over previous graph neural networks trained on single graphs. We also employed model explanation techniques to assess both node and edge feature importance. Our results showed that EMGNN leveraged the complementary information from different graph layers and omics features to predict cancer genes. Importantly, we found that cancer genes that have conflicting predictions based on different single graphs or are missed by previous state-of-the-art predictors, can be recovered using EMGNN. This demonstrates the robustness of EMGNN predictions by joint modeling the different graphs.

The EMGNN model can be viewed as a data-driven, gradient-enabled integration method for multiple graphs. Since all PPI networks share the same type of nodes and edges, EMGNN currently integrates homogeneous, undirected graphs; however, our framework can be extended to include hierarchical or heterogeneous graphs and to perform cross-data modality integration with diverse node and edge types. Another future direction is to extend our framework for multilabel classification tasks, including the fine-grained classification of cancer types based on their tissue of origin or other distinctions. Such expansion is of high clinical importance and straightforward to implement using our EMGNN framework when properly labeled data are available in the future.

## Supplementary Material

btad643_Supplementary_Data

## Data Availability

The extraction of node features was performed using data obtained from TCGA, accessible at https://www.cancer.gov/ccg/access-data. The node labels were acquired from NCG, available at https://genomebiology.biomedcentral.com/articles/10.1186/s13059-018-1612-0, as well as Cosmic CGC, accessible via https://pubmed.ncbi.nlm.nih.gov/30293088/. The source code for this work is publicly available on GitHub at https://github.com/zhanglab-aim/EMGNN.
