# Supplementary material for: Explainable Multilayer Graph Neural Network for cancer gene prediction
Source: Bioinformatics. 2023 Oct 20;39(11):btad643. doi: 10.1093/bioinformatics/btad643 (PMC10636280; doi:10.1093/bioinformatics/btad643)
Supplement: btad643_Supplementary_Data [file btad643_supplementary_data.pdf]

Supplementary Material: Explainable Multilayer Graph Neural Network for Cancer Gene Prediction

1 Ablation Studies

Ablation Study: Assessing the Importance of Node Features and Graph Structure in EMGNN for Cancer Gene Prediction. We conducted a comprehensive ablation study aimed at answering two key questions: First, how does EMGNN handle perturbations in node features and graph structure? Secondly, are both node features and graph structure essential for predicting cancer genes effectively? Our study involved various input perturbations, including the removal of multi-omics node features, edges, and the addition of random or constant vectors. We report the results in Table 1 Notably, we observe that EMGNN's performance declined when subjected to random or all-one node features, underscoring the informativeness and relevance of the node features derived from the TCGA consortia. These node features provide critical insights into cancer pathophysiology and gene pathogenicity prediction. Regarding edge ablations, where we randomly removed 20% and 40% of edges in each PPI network, we found that the removal of edges had also an impact on EMGNN's performance. Overall, our findings highlight EMGNN's effective utilization of both node features and edges to achieve accurate predictions, emphasizing their complementary roles in the model's predictive capabilities.

Table 1: Test AUPRC and standard deviation of EMGNN(GCN) for different input perturbations methods across three different runs.

Method	CPDB	Multinet	PCNet	STRING-db	Iref	Iref(2015)
**Random Features**	0.703 ± 0.001	0.727 ± 0.002	0.615 ± 0.009	0.745 ± 0.002	0.674 ± 0.001	0.697 ± 0.005
**All-one Features**	0.726 ± 0.002	0.769 ± 0.001	0.657 ± 0.010	0.779 ± 0.010	0.710 ± 0.015	0.725 ± 0.013
**Edge Removal(0.2)**	0.800 ± 0.007	0.841 ± 0.016	0.746 ± 0.017	0.841 ± 0.009	0.796 ± 0.005	0.786 ± 0.011
**Edge Removal(0.4)**	0.795 ± 0.004	0.834 ± 0.009	0.743 ± 0.003	0.828 ± 0.004	0.790 ± 0.012	0.802 ± 0.006

Ablation study: Impact of number of GNN layers. In this ablation study, we study the impact of varying the number of GNN layers on EMGNN(GCN)'s predictive performance. We evaluated the model's performance on multiple PPI networks, including CPDB, Multinet, PCNet, STRING-db, Iref, and Iref(2015), while systematically altering the number of GNN layers from 1 to 6. As observed in the results in Table 2, increasing the number of layers from 1 to 3 leads to a notable improvement in AUPRC, suggesting that a deeper architecture can capture more intricate gene-gene interactions within this network. When employing just one GNN layer, the model exhibited limitations in capturing information from more distant neighbors in the graph. This limitation can be detrimental as it fails to consider important interactions that span multiple nodes away. On the other hand, when a large number of GNN layers, the model's complexity increases while there is an oversmoothing problem. In our case, employing three GNN layers provided the best trade-off in most cases, enabling effective information propagation while maintaining model efficiency and generalization.

Table 2: Test AUPRC of EMGNN(GCN) with varying numbers of GNN layers.

GNN layers	CPDB	Multinet	PCNet	STRING-db	Iref	Iref(2015)
**1**	0.775 ± 0.008	0.827 ± 0.008	0.727 ± 0.001	0.823 ± 0.004	0.760 ± 0.002	0.752 ± 0.006
**2**	0.789 ± 0.005	0.853 ± 0.009	0.752 ± 0.002	0.854 ± 0.007	0.805 ± 0.003	0.795 ± 0.008
**3**	0.809 ± 0.006	**0.854** ± 0.007	**0.761** ± 0.001	**0.856** ± 0.002	**0.822** ± 0.002	0.800 ± 0.010
**6**	**0.817** ± 0.006	0.827 ± 0.007	0.749 ± 0.003	0.834 ± 0.005	0.804 ± 0.007	**0.810** ± 0.007

Ablation study: Impact of Edge Confidence Threshold. In this ablation study, we investigate the performance of our EMGNN model across a spectrum of edge confidence threshold levels for the STRING-db network. Our results in Table 3 reveal that performance variations, while present, remain relatively modest, underscoring the model's resilience across diverse threshold settings. Notably, we achieved our most compelling performance when employing the highest confidence

threshold of 95%, where only the most crucial and reliable edges are retained in the network. This ensures that the resulting PPI graph is characterized by a higher degree of confidence in the interactions it represents.

Table 3: EMGNN(GCN) Performance with varying Edge Confidence Threshold on STRING-db.

Threshold	Accuracy	AUPRC	AUROC
**0.5**	0.890 ± 0.002	0.860 ± 0.002	0.932 ± 0.01
**0.65**	0.888 ± 0.002	0.858 ± 0.004	0.931 ± 0.002
**0.75**	0.883 ± 0.005	0.856 ± 0.003	0.928 ± 0.002
**0.85**	0.876 ± 0.002	0.857 ± 0.005	0.929 ± 0.001
**0.95**	0.861 ± 0.007	0.885 ± 0.012	0.921 ± 0.006

Incorporation of Tissue Networks. In this experiment, we explored various tissue-specific networks instead of PPI networks. The utilization of gene-gene tissue networks holds the promise of unraveling tissue-specific complexities in cancer genomics, offering a more nuanced and contextually relevant understanding of gene interactions within distinct biological environments. Specifically, we incorporated biological networks derived from three distinct tissue types: the adrenal cortex, blood, and adipose tissues from NetWAS [Greene et al., 2015]. We use as training, validation, and test sets the labeled genes from the CPDB dataset. We present the results in Table 4. When considering individual tissues, we observe similar performance with the PPI networks, indicating that the tissue-specific information is also important for the cancer-gene prediction task. Furthermore, the integration of multiple networks proved to be a valuable strategy in this case as well. When combining the adrenal cortex and adipose tissue networks, we observed improvements in the predictions, highlighting the complementary nature of tissue-specific networks. This synergistic effect continued with the addition of the blood network, resulting in the highest accuracy, AUPRC, and AUROC values achieved when all three tissue networks were combined.

Table 4: EMGNN(GCN) Performance Across Tissue-Specific Networks.

Threshold	Accuracy	AUPRC	AUROC
Adrenal Cortex	0.748 ± 0.007	0.802 ± 0.017	0.795 ± 0.025
Adipose Tissue	0.753 ± 0.013	0.797 ± 0.014	0.798 ± 0.013
Blood	0.692 ± 0.008	0.787 ± 0.017	0.775 ± 0.008
Adrenal Cortex, Adipose Tissue	0.751 ± 0.007	0.818 ± 0.008	**0.821** ± 0.012
Adrenal Cortex, Blood	0.750 ± 0.004	0.810 ± 0.006	0.805 ± 0.001
Adipose Tissue, Blood	0.723 ± 0.008	0.804 ± 0.007	0.807 ± 0.008
Adrenal Cortex, Adipose Tissue, Blood	**0.767** ± 0.004	**0.821** ± 0.002	0.819 ± 0.004

2 Experimental Details

Baselines. We compared the performance of EMGNN against several baseline methods for predicting cancer genes. The baseline methods include traditional machine learning algorithms, as well as state-of-the-art graph-based models and gene prioritization methods. Specifically, we compare against the following baselines: 1)Random, which randomly assigns a probability of being a cancer gene to each gene. 2) 20/20+ [Tokheim et al., 2016], which is a machine learning method that combines features extracted from gene expression data with known cancer genes and non-cancer genes. 3) MutSigCV [Lawrence et al., 2013], which is a statistical method for identifying significantly mutated genes in cancer samples. 4) HotNet2 diffusion [Leiserson et al., 2015], which uses heat diffusion to detect clusters of genes with significant mutations. 5) DeepWalk+features RF, which utilizes both network topology using random walks and additional features to predict cancer genes. 6) PageRank which is applied to prioritize genes as potential cancer genes based on their network centrality [Page et al., 1998]. 7) GCN without omics, which uses a Graph Convolutional Network without incorporating omics data for predicting cancer genes. 8) DeepWalk + SVM, which combines DeepWalk with a Support Vector Machine (SVM) classifier to predict cancer genes. 9) RF, which uses a random forest classifier on the multi-omics data. 10) MLP, which uses a multi-layer perceptron on the multi-omics data. 11) EMOGI [Schulte-Sasse et al., 2021], which combines multi-omics data with a graph convolutional neural network to predict cancer genes. 12) EMOGI(Majority Vote), which combines the predictions from multiple models trained on different PPI networks. 13) EMOGI [Hong et al., 2022], which employs an optimized implementation of EMOGI.

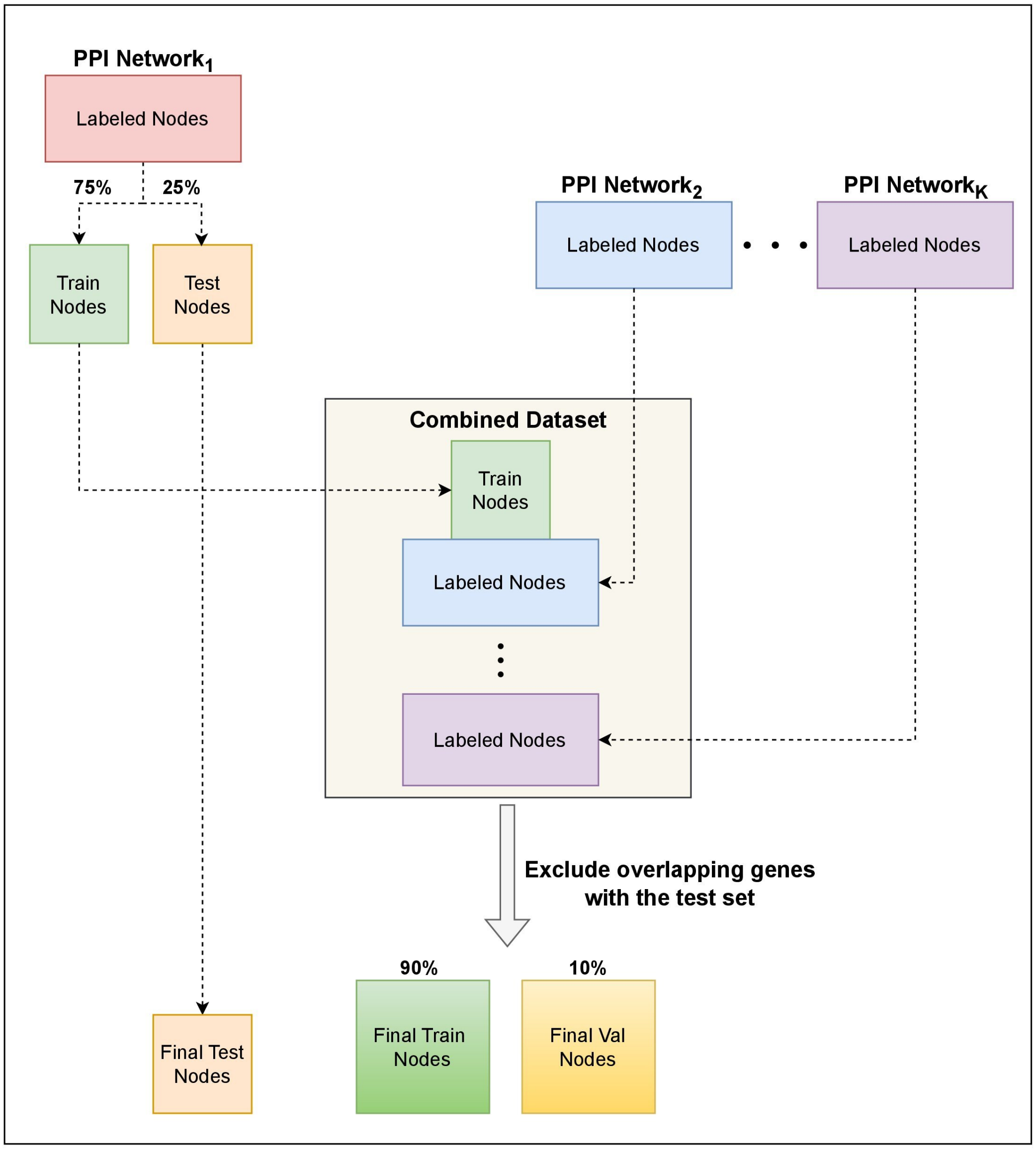

Figure 1: An illustration of the process of adding new protein-protein interaction (PPI) networks in the training procedure. Starting with an initial PPI network (PPI Network 1), the nodes are divided into training and test sets. To expand the training set, additional PPI networks are introduced, with their nodes added to the existing training set while keeping the test set fixed from just the first network.

Hyperparameters We highlight the key hyperparameters of our model, along with the optimal values we ascertained: For the learning rate, we explored values between 0.001 and 0.01, with an optimal rate of 0.005. For the weight decay, we searched values between 1e-5 and 1e-3, with an optimal weight decay of 5e-4. For the hidden units in the GNN, we searched from 32 to 128, with an optimal value of 64. For the attention heads, we searched from 1 to 8, with one head performing the best. For the dropout rate, we searched from 0.3 to 0.7, with an optimal rate of 0.5. For the number of layers, we searched from 1 to 6 layers, with 3 layers performing the best. These optimal values were selected based on the best AUPRC in the validation set.

Table 5: Statistics and properties for the PPI Networks.

**PPI Network**	**Properties**						
	Number of Nodes	Number of Edges	Positive Nodes	Negative Nodes	Train Nodes	Val Nodes	Test Nodes
**CPDB**	13627	518005	796	2187	2013	224	746
**Multinet**	14398	233532	790	3709	3036	338	1125
**PCNet**	19781	5469229	859	5483	4280	476	1586
**STRING-db**	13179	686278	783	2415	2158	240	800
**Iref**	17013	760150	836	4056	3302	367	1223
**Iref(2015)**	12129	195747	785	1973	1861	207	690

References

Greene, C. S., Krishnan, A., Wong, A. K., Ricciotti, E., Zelaya, R. A., Himmelstein, D. S., Zhang, R., Hartmann, B. M., Zaslavsky, E., Sealfon, S. C., et al. (2015). Understanding multicellular function and disease with human tissue-specific networks. *Nature genetics*, 47(6):569–576.

Hong, C., Cao, Q., Zhang, Z., Tsui, S. K.-W., and Yip, K. Y. (2022). Reusability report: Capturing properties of biological objects and their relationships using graph neural networks. *Nature Machine Intelligence*, 4(3):222–226.

Lawrence, M. S., Stojanov, P., Polak, P., Kryukov, G. V., Cibulskis, K., Sivachenko, A., Carter, S. L., Stewart, C., Mermel, C. H., Roberts, S. A., et al. (2013). Mutational heterogeneity in cancer and the search for new cancer-associated genes. *Nature*, 499(7457):214–218.

Leiserson, M. D., Vandin, F., Wu, H.-T., Dobson, J. R., Eldridge, J. V., Thomas, J. L., Papoutsaki, A., Kim, Y., Niu, B., McLellan, M., et al. (2015). Pan-cancer network analysis identifies combinations of rare somatic mutations across pathways and protein complexes. *Nature genetics*, 47(2):106–114.

Page, L., Brin, S., Motwani, R., and Winograd, T. (1998). The pagerank citation ranking: Bring order to the web. Technical report, Technical report, stanford University.

Schulte-Sasse, R., Budach, S., Hnisz, D., and Marsico, A. (2021). Integration of multiomics data with graph convolutional networks to identify new cancer genes and their associated molecular mechanisms. *Nature Machine Intelligence*, 3(6):513–526.

Tokheim, C. J., Papadopoulos, N., Kinzler, K. W., Vogelstein, B., and Karchin, R. (2016). Evaluating the evaluation of cancer driver genes. *Proceedings of the National Academy of Sciences*, 113(50):14330–14335.
